# Constructing a prediction model for physiological parameters for malnutrition in hemodialysis patients

**DOI:** 10.1038/s41598-019-47130-7

**Published:** 2019-07-24

**Authors:** Yu-Tsung Tsai, Feng-Jung Yang, Hong-Mau Lin, Jiang-Chou Yeh, Bor-Wen Cheng

**Affiliations:** 1 Department of Industrial Engineering and Management, National Yunlin University of Science and Technology, Douliu City, Yunlin County, Taiwan; 2 Department of Internal Medicine, National Taiwan University Hospital Yun-Lin Branch, Douliu City, Yunlin County, Taiwan; 3 Graduate Institute of Clinical Medicine, College of Medicine, National Taiwan University, Taipei City, Taiwan; 4 Institute of Health Policy and Management, National Taiwan University, Taipei City, Taiwan; 5 Department of Internal Medicine and Department of Medical Genetics, National Taiwan University Hospital, Taipei City, Taiwan; 6 Department of Surgery, National Taiwan University Hospital Yun-Lin Branch, Douliu City, Yunlin County, Taiwan

**Keywords:** Preclinical research, End-stage renal disease, Nephrosclerosis

## Abstract

A retrospective analysis of the improvement in the health condition of patients undergoing hemodialysis was done to understand the important factors that can affect malnutrition in these patients. In this study, data from patients who underwent hemodialysis between 2010 and 2015 in a regional hospital in Yunlin County were collected from the Taiwan Society of Nephrology-Kidney Transplantation database. A total of 1049 medical records from 300 patients with age over 20 and underwent hemodialysis were collected for this study. A decision tree C5.0 and logistic regression were used to identify 40 independent variables, as well as the association of the dependent variable albumin. Then, the C5.0 decision tree, logistic regression, and support vector machine (SVM) methods were applied to find a combination of factors that contributed to malnutrition in patients undergoing hemodialysis. Predictive models were established. Finally, a receiver operating characteristic curve and confusion matrix was used to evaluate the standard of performance of these models. All analytical methods indicated that “age” was an important factor. In particular, the best predictive model was the SVM-model 4, with a training accuracy rate of 98.95% and test accuracy rate of 66.89%, identified that “age” and 15 other important factors were the most related to hemodialysis. The findings of this study can be used as a reference for clinical applications.

## Introduction

**Figure 1:**
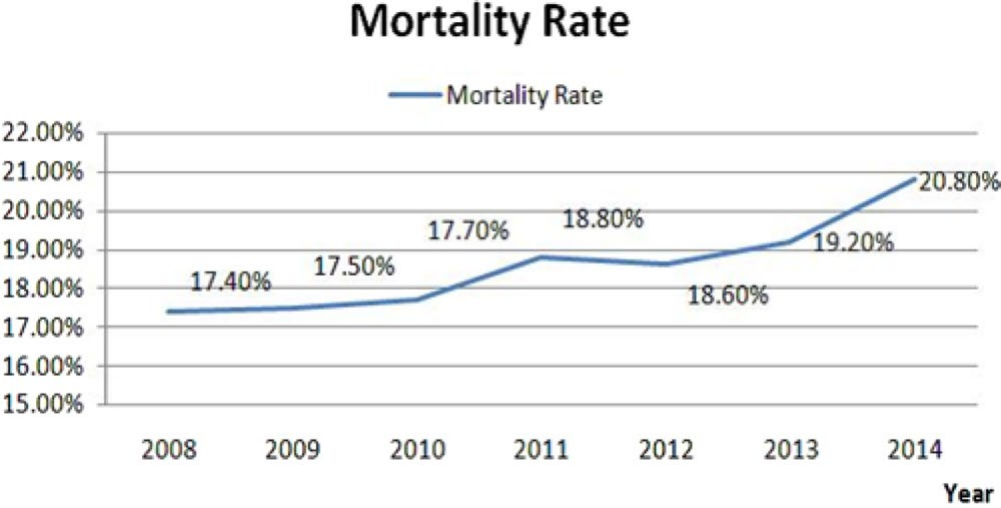
Mortality rate for nephritis, nephrotic syndrome, and nephropathy in the last seven years.

Since 2001, Taiwan had the highest incidences of end-stage renal disease (ESRD) in the world and the second highest in 2009. Statistics, published by Ministry of Health and Welfare from 2008 to 2014, cites that nephritis, nephrotic syndrome and nephropathy were among the top ten causes of death in Taiwan. Also, the number of deaths and mortality rate have been increasing annually, as shown in Fig. 1^1^.

Patients undergoing long-term hemodialysis are prone to protein-energy malnutrition (PEM) conditions. Studies have shown that approximately 18–70% of patients undergoing hemodialysis have PEM complications. The proportion of malnutrition in elderly dialysis patients is even higher^2^. In the current study which uses the Taiwan Society of Nephrology-Kidney Transplantation (TSN-KiDiT) database, data were retrospectively collected from 300 patients aged above 20 and had undergone hemodialysis in National Taiwan University Hospital, Yun-Lin Branch between 2010 and 2015. Data from a total of 1049 medical records were collected. Data mining techniques were applied to identify factors related to malnutrition in patients undergoing hemodialysis. Also, all relevant factors related to malnutrition were identified in patients undergoing hemodialysis with and without malnutrition. These findings were provided to the physicians of the hospital as a reference for clinical treatment, thereby improving the nutritional conditions of patients undergoing hemodialysis. The objectives of the current study were two-fold. First, we aimed to evaluate the significance of the correlation between various factors in the database and the dependent variable ‘albumin’. Second, we aimed to identify the best predictive model of factors affecting malnutrition in patients undergoing hemodialysis, and to use this model to provide reference values to improve prediction and treatment of malnutrition in these patients.

## Results

### Descriptive statistics

A total of 1,049 data items from 300 patients were analyzed in the current study. The mean age of patients was 64.55 years old (standard deviation 13.71 years). Among them, most patients were 67–73 years old, which accounted for 19% of the total. The patient cohort also mainly consisted of men, who accounted for 52.6% of patients. Furthermore, 53.8% of patients had a primary systemic disease and 74.2% had an autologous arteriovenous fistula. A total of 51.5% of the patient cohort was malnourished. With regards to the statistical analysis, there was no significant difference in the mean age between the two groups of patients.

### The selection of training sample group and test sample group

The current study was based on the study conducted by^3^. The k-fold cross-validation method, where K = 10 as the sample division, was applied to this study. Each fold of data included 104 cases, (i.e., 1,049 data items/10 = 104 cases). The remaining nine samples were added into any fold group in a random and non-repeated order. In the study sample, according to the dependent variable’malnutrition’, the cases were divided into ‘non-malnourished’ (513 cases) and ‘malnourished’ (536 cases). The cases were placed into each sample group according to the principle of proportionality.

### Pearson correlation

In this study, the Pearson product-moment correlation coefficient was used to investigate the correlation between a total of 40 factors including age, Gender, Year of treatment, Primary disease type, Type of fistula, comorbidities 1, comorbidities 2, A.S.T[GOT], A.L.T[GPT], Alkaline-P, Cholesterol, Trigleceride, Glucose, W.B.C, R.B.C, Hbc, Hct, MCV, Platelet, Fe, TIBC, Ferritin, Transferritin saturation, pre-dialysis body weight, Post-dialysis weight, Weight loss from dialysis, Prior- dialysis blood urea nitrogen (BUN) level, Post-dialysis blood urea nitrogen (BUN) level, Creatinine, Uric acid, K, Calcium, P, URR, Kt/V(Gotch), NPCR, TAC urea, Kt/V, Dehydration from ultrafiltration, PTH and serum albumin, i.e. the nutrition index of hemodialysis patients. The results showed that 32 factors were significantly correlated with serum albumin. The correlation between various factors and the presence of malnutrition in hemodialysis patients are shown in Appendix 1.

### Multiple regression analysis

**Figure 2:**
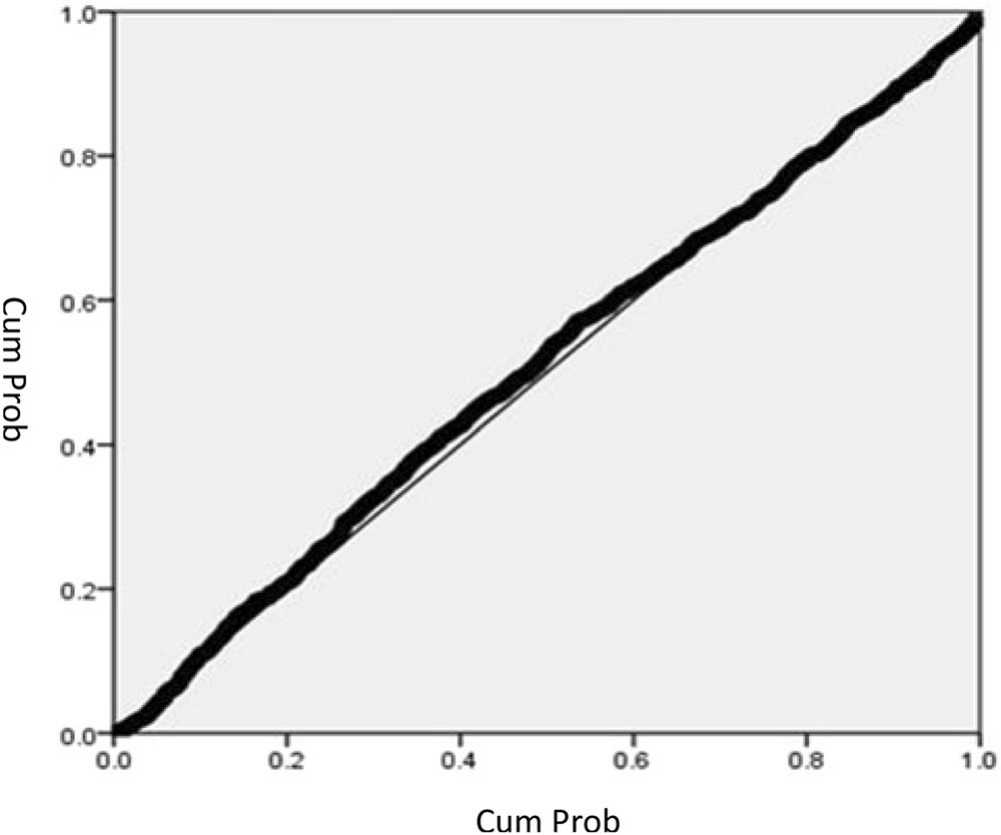
Normal probability P-P plot of multivariate regression analysis results were included.

**Figure 3:**
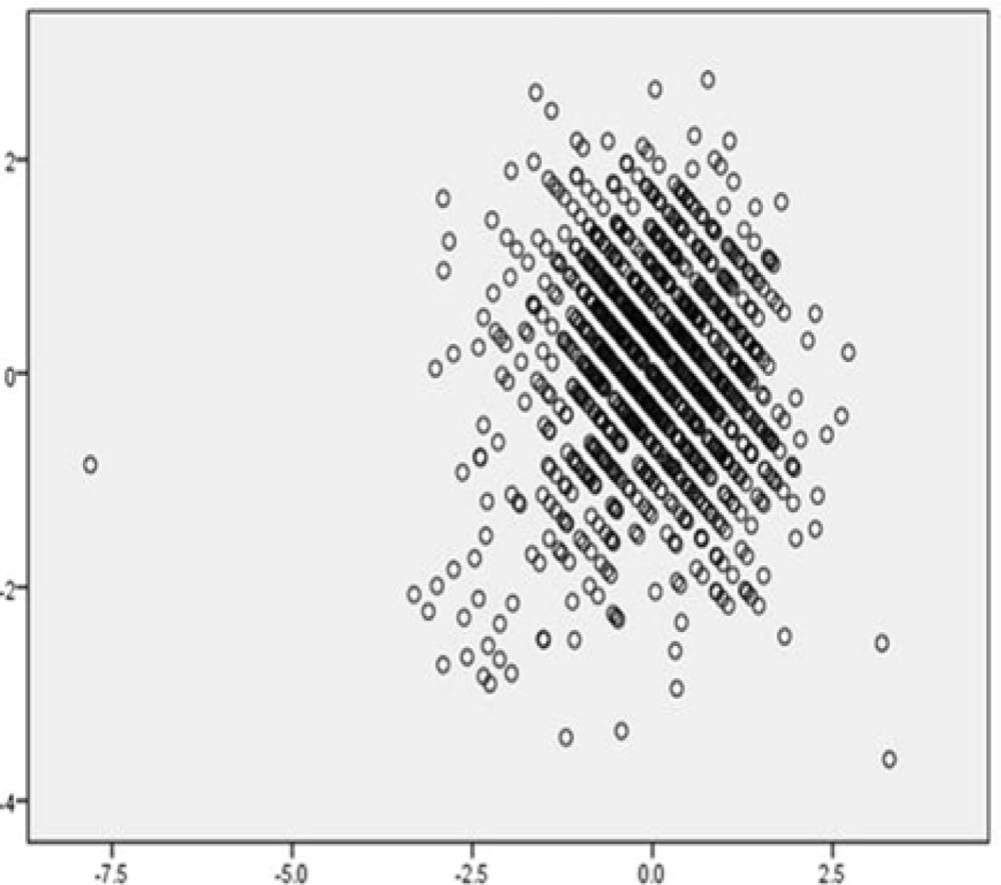
Scatter plot of multiple regression analysis results were included.

In this study, multiple regression analysis was applied to understand the effect of serum albumin, i.e. the nutrition index of hemodialysis patients, on a total of 35 factors including age, year of treatment, A.S.T[GOT], A.L.T[GPT], Alkaline-P, Cholesterol, Trigleceride, Glucose, W.B.C, R.B.C, Hbc, Hct, MCV, Platelet, Fe, TIBC, Ferritin, Transferritin saturation, pre-dialysis body weight, Post-dialysis weight, Weight loss from dialysis, Prior- dialysis blood urea nitrogen (BUN) level, Post-dialysis blood urea nitrogen (BUN) level, Creatinine, Uric acid, K, Calcium, P, URR, Kt/V(Gotch), NPCR, TAC urea, Kt/V, Dehydration from ultrafiltration and iPTH, using a “stepwise multiple regression analysis.” The results showed that 14 factors had significant explanatory power for serum albumin. The regression analysis results of each factor and serum albumin for hemodialysis patients are shown in Appendix 2, Fig. 2, the observed values of the samples were very close to the estimated normal distribution and Fig. 3, the observed values of the samples conformed to the assumptions of normality and homogeneity of variance.

### The results of the C5.0 Decision Tree model

The optimal allocation of sample groups for the C5.0 decision tree model and cross-validation method were in training sample groups and one test sample group. After allocations had been completed, training and testing sample groups were entered into the IBM SPSS Modeler software to construct the C5.0 Decision Tree model. This study obtained the accuracy of sample classifications from the 10-fold cross-validation of 10 sample groups^4^. Group 8, in particular, produced the best results. A total of 28 of the following important and significant factors were screened out: age, hemoglobin level (Hbc), total iron binding capacity(TIBC), creatinine, K, type of fistula (CHDCAPTYPE), patient weight prior to dialysis, triglyceride level, weight loss after dialysis, sex, cholesterol, alanine aminotransferase (ALT)/glutamic-pyruvic transaminase(GPT), normalized protein catabolic rate, year of treatment, white blood cell count (WBC), platelet level, ferritin level, hematocrit (Hct), parathyroid hormone (PTH), transferrin saturation, aspartate aminotransferase (AST), urea reduction ratio (URR), red blood cell count (RBC), patient weight post-dialysis, calcium, mean corpuscular volume (MCV), glucose, and phosphorus(P).

### Results of logistic regression

In this study, the results of the classification accuracy were obtained by the 10-fold cross-validation of 10 sample groups. Among these, the results of Group 10 was the best. A total of 15 important and significant factors were screened out, namely, the following: age, HBC, Creatinine, type of fistula (CHDCAPTYPE), potassium (K), year of treatment, primary disease type, AST, TIBC, calcium, platelet, MCV, WBC, post-dialysis blood urea nitrogen (BUN) level, and Hct.

### SVM modeling and results

In this study, the factors entered into the SVM model were divided according to the following five ways:

1. SVM model 1:
  All 40 factors were entered, and Group 6 produced the best result. A total of 20 important correlation factors, which included age etc. were screened out.
2. SVM model 2:
  The 28 factors identified by C5.0 decision tree were entered, and Group 5 produced the best result. A total of 14 important correlation factors, which included age etc. were screened out.
3. SVM model 3:
  The 15 factors identified by logistic regression were entered, and Group 5 produced the best result. A total of 8 important correlation factors, which included age etc. were screened out.
4. SVM model 4:
  The 32 factors identified by Pearson product-movement correlation were entered, and Group 5 produced the best result. A total of 15 important correlation factors, which included age etc. were screened out.
5. SVM model 5:

The 14 factors identified by multiple regression analyses were entered, and Group 5 produced the best result. A total of 6 important correlation factors, which included age etc. were screened out.

### Performance evaluation of each model

In this study, the confusion matrix and the ROC curve were applied to evaluate and compare the performance of seven different models. The confusion matrix of each model was applied to identify the best model. The average accuracy and standard deviation of the 10-fold cross-validation model, as well as the aggregated results of the performance of each prediction model, were evaluated based on the screening factors were shown in Appendix 3.

## Discussion

1. The models were evaluated for medical application by physicians in the case hospital. The best predictive model in this study was evaluated by the physicians. The input of 32 correlation factors, which were identified by the Pearson product-movement correlation, into the SVM model for data mining produced 15 important correlation factors that were worthy of clinical interpretation and application. Also, this result corroborated with previous literature^5–7^.
2. The best predictive model of this study was SVM Model 4, which had an accuracy rate of 73.01%, average training accuracy of 98.95%, average test accuracy of 66.89%, and a standard deviation of 2.2%. If a mixed model matrix was applied, the accuracy rate reached 73.01%. A total of 15 important correlation factors were identified, which in a descending order of importance (from highest to lowest) were age, type of fistula(CHDCAPTYPE), gender, cholesterol, TIBC, uric acid, creatinine, alkaline-P, platelet count, body weight post-dialysis, body weight prior-dialysis, Hbc, Hct, dehydration from ultrafiltration, and phosphorus (P).
3. Finally, Pearson Correlation ‘Multiple Regression Analysis’ the C5.0 decision tree and logistic regression were applied to find the nine common factors, which were age, year of treatment, type of fistula (CHDCAPTYPE), Hbc, Hct, TIBC, creatinine level, potassium (K), and calcium.

## Methods

### Research framework and data collection

This study met the research ethics guidelines and was approved by the Ethics Committee of the National Taiwan University Hospital on November 6, 2015. The case number is 201510041RINA. After the approval, patient data were collected. A total of 18,000 data items were collected from January 2010 to December 2015, using the case hospital’s patient database of those undergoing hemodialysis. Patient data were encoded and combined. The inclusion and exclusion criteria were as outlined below.

1. Inclusion criteria:

- Patients must be over than 20 years old.
- Patients must have started hemodialysis at the target hospital.

(2) Exclusion criteria:

- Patients who started hemodialysis at non-target hospitals.
- Patients who were transferred to a different hospital during the treatment period, or who underwent a change in treatment method (e.g., from hemodialysis to peritoneal dialysis, who underwent a kidney transplant).
- Patients undergoing peritoneal dialysis.

Office Excel 2007 Visual Basic for Application (VBA) was used for data review and screening. Sample data were normalized and missing values were removed. Data preprocessing was conducted as outlined below. In data mining, data preparation and model construction required the processing of a large quantity of data. In this study, there were two parts of data processing.

1. Missing values: For data samples, if data were either missing or incomplete when it was uploaded to the database, it was removed for the purpose of this study.
2. Normalization: Before input into the SVM, data were normalized and converted into a format, which could be read by the SVM, using the equation below (3.1)

3.1 $${V}_{i}^{\ast }=\frac{Vi-Vmin}{Vmax-Vmin}$$

in which, Vi* = Normalized value, Vi = Actual Value, Vmin = The minimum V value, and Vmax = The maximum V value. Data were integrated using VBA macro language in Office Excel 2007. Demographic data and blood sample data were integrated, and check-up data sheets were defined by the field name ‘Patient ID’in the data integration process. This process enabled us to understand the test results of all the patients. In conclusion, a total of 300 patients undergoing hemodialysis, and 1,049 data items, were identified as being suitable. C5.0 decision tree and logistic regression were applied to identify the optimal combination of influential factors, which resulted in malnutrition in patients undergoing hemodialysis. This result was used to construct the first-stage predictive model. Then, SVM was applied to establish the second-stage predictive model. Finally, the performance of each predictive model was compared and discussed, in order to select the best predictive model.

### Variable collections

The variables related to malnutrition in patients undergoing hemodialysis were selected from the database. A total of 40 independent variables were selected, which were compiled into tables.

### Support vector machine parameter settings

In this study, IBM SPSS Modeler was used to construct the SVM models. In this study, parameter settings were obtained using the grid search method applied by^8^. The method used is detailed below:

Step 1: Variable conversion.

The 40 factors (independent variables) were used as input. The prediction of whether patients undergoing hemodialysis were malnourished or not was the output.

Step 2: Model construction.

1. The training sample group was used to train the network model and obtain the best parameter settings.
2. The ‘Stop Conditions calculated, i.e., the condition to stop training the network model. This result was also the most optimal parameter settings for the model.
3. After the most optimal parameter settings had been determined for the SVM classification model, test samples were entered into the model to evaluate its accuracy rate.

Exhibit 3: Accuracy rates of training and testing of the grid search method, as shown in Appendix 4.

### Construction of K-fold cross-validation method

The K-fold cross-validation method was applied in the current study. In the K-fold cross-validation method the data samples were randomly divided into K sample groups. The number of samples in each sample group were the same; the K-1 sample group is used as the training sample and the rest were testing samples. Model constructions were repeated K times. This method can effectively reduce predictive error and variation caused by random classification^9^. In the current study, data samples were randomly divided into ten samples, and the predictive error and variation were minimum when K = 10 as the sample division of the study.

### Construction of the logistic regression model

The dependent variable of this study was the patient was malnourished. Logistic regression was also used to test whether there was a significant correlation between independent variables and the dependent variable in this model.

### Construction of the C5.0 decision tree model

In this study, the IBM SPSS Modeler data mining software was used to build the C5.0 decision tree model to analyze the patients undergoing hemodialysis, using the database of the case hospital.

### Pearson correlation

In this research, we used a Pearson correlation analysis to understand the correlation between 40 independent variables and the dependent variable serum albumin in the TSN-KiDiT database after data integration.

### Multiple regression analysis

In this study, multiple regression analysis was used to evaluate the overall predictive ability and descriptive capacity of independent variables on dependent variables. In the current study, there was a degree of correlation between the 40 variables. Thus, multiple regression analysis was applied to understand the effect of serum album level on the significance and descriptive capacity of the 40 independent variables in the TSN-KiDIT database.

### Evaluation of model performance

In the current study, a confusion matrix and receiver operating characteristic (ROC) curve were used to evaluate the accuracy of the two models. The distinction of the performance of the model was based on a previous study^10^. First, the construction of confusion matrix was recommended, as shown in Appendix 5.

TP: The proportion of actual recurrence to correct prediction of recurrence.

FP: The proportion of actual recurrence to incorrect prediction of non-recurrence

FN: The proportion of actual non-recurrence to incorrect prediction of recurrence. TN: The proportion of actual non-recurrence to correct prediction of non-recurrence. Next, the TP, FP, FN, and TN were used as a basis to test for its accuracy, and construct the ROC curve, which demonstrated the sensitivity and specificity of the models. The equations used are shown in Appendix 6 ^5–7^^,^^11–16^.

## Supplementary information

Supplementary Fig1

Supplementary Fig2

Supplementary Fig3

APPENDIX
