# Supplementary figures and images for: Constructing a prediction model for physiological parameters for malnutrition in hemodialysis patients
Source: Sci Rep. 2019 Jul 24;9:10767. doi: 10.1038/s41598-019-47130-7 (PMC6656719; doi:10.1038/s41598-019-47130-7)

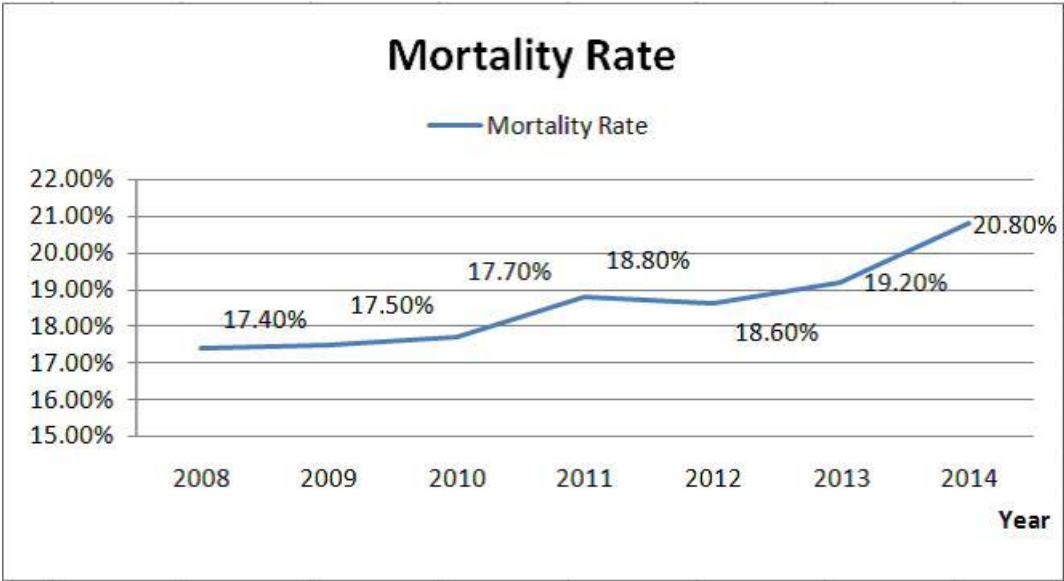

Supplement: Supplementary file 1 — Supplementary Fig1 [file 41598_2019_47130_MOESM1_ESM.pdf]

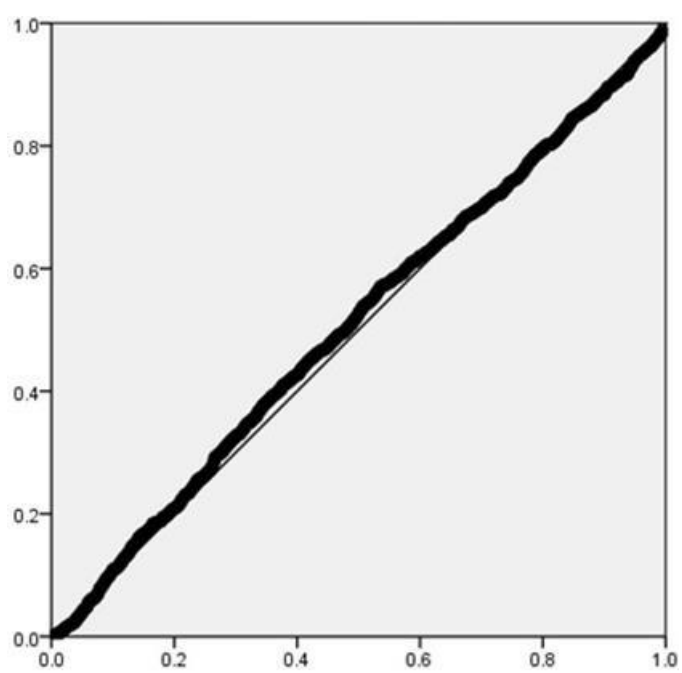

Supplement: Supplementary file 2 — Supplementary Fig2 [file 41598_2019_47130_MOESM2_ESM.pdf]

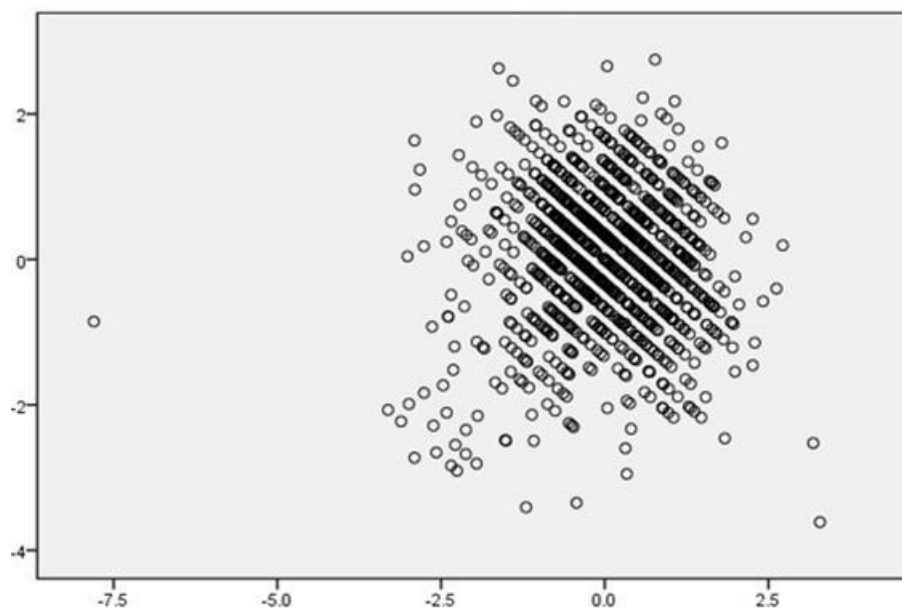

Supplement: Supplementary file 3 — Supplementary Fig3 [file 41598_2019_47130_MOESM3_ESM.pdf]
