# Supplementary material for: Constructing a prediction model for physiological parameters for malnutrition in hemodialysis patients
Source: Sci Rep. 2019 Jul 24;9:10767. doi: 10.1038/s41598-019-47130-7 (PMC6656719; doi:10.1038/s41598-019-47130-7)
Supplement: Supplementary file 4 — APPENDIX [file 41598_2019_47130_MOESM4_ESM.docx]

**Constructing a prediction model for physiological parameters for malnutrition in hemodialysis patients**

**Yu-Tsung Tsai^1^, Feng-Jung Yang^2*^, Hong-Mau Lin^3^, Jiang-Chou Yeh^4^, Bor-Wen Cheng^5^**

**^1^Department of Industrial Engineering and Management National Yunlin University of Science and Technology Yunlin, Taiwan.**

**^2*^Department of Internal Medicine National Taiwan University Hospital, Yun Lin Branch Yunlin, Taiwan.**

**Graduate Institute of Clinical Medicine, College of Medicine, National Taiwan University, Taipei, Taiwan.**

**Institute of Health Policy and Management, National Taiwan University.**

**Department of Internal Medicine, National Taiwan University Hospital Yun Lin Branch, Douliu, Taiwan.**

**^3^Consultant of Superintendent Room National Taiwan University Hospital Yun Lin Branch, Yunlin, Taiwan.**

**^4^Department of Industrial Engineering and Management National Yunlin University of Science and Technology Yunlin, Taiwan.**

**^5^Department of Industrial Engineering and Management National Yunlin University of Science and Technology Yunlin, Taiwan.**

**^*^Corresponding author’s email address: QQ929010@gmail.com**

**APPENDIX 1**

|  | Dependent Variables：Albumin | |
| --- | --- | --- |
|  | r | p |
| Age | -0.305** | 0.00 |
| Type of fistula | -0.302** | 0.00 |
| Creatinine | 0.422** | 0.00 |
| Gender | -0.142** | 0.00 |
| Year of treatment | -0.095** | 0.02 |
| A.S.T[GOT] | -0.185** | 0.00 |
| A.L.T[GPT] | -0.137** | 0.00 |
| Alkaline-P | -0.139** | 0.00 |
| Cholesterol | 0.138** | 0.00 |
| Triglyoeride | 0.095** | 0.02 |
| Glucose | -0.079* | 0.01 |
| R.B.C | 0.221** | 0.00 |
| Hbc | 0.242** | 0.00 |
| Hct | 0.206** | 0.00 |
| MCV | -0.118** | 0.00 |
| Platelet | 0.08** | 0.00 |
| TIBC | 0.221** | 0.00 |
| Ferritin | -0.134** | 0.00 |
| Tranferritin saturation | -0.114** | 0.00 |
| pre-dialysis body weight | 0.119** | 0.00 |
| Post-dialysis weight | 0.157** | 0.00 |
| Prior-dialysis blood urea nitrogen (BUN) level | 0.108** | 0.00 |
| Post-dialysis blood urea nitrogen (BUN) level | 0.116** | 0.00 |
| Uric acid | 0.104** | 0.01 |
| K | 0.246** | 0.00 |
| Calcium | 0.198** | 0.00 |
| P | 0.165** | 0.00 |
| Kt/V(Gotch) | -0.062* | 0.04 |
| NPCR | 0.081** | 0.00 |
| TAC urea | -0.119** | 0.00 |
| Dehydration from ultrafiltration | 0.125** | 0.00 |
| PTH | 0.078* | 0.01 |

***p<0.05 , **p<0.01**

**APPENDIX 2**

| Dependent Variables：Albumin | | | | | |
| --- | --- | --- | --- | --- | --- |
|  | Beta  Estimate | Standard Error | Beta | T | P |
| Constant | 2.844 | 0.853 |  | 3.333 | 0.001 |
| Calcium | 0.216 | 0.054 | 0.106 | 4.031 | 0.000** |
| Hbc | 0.176 | 0.029 | 0.632 | 6.140 | 0.000** |
| K | 0.086 | 0.015 | 0.160 | 5.716 | 0.000** |
| Creatinine | 0.035 | 0.005 | 0.257 | 7.288 | 0.000** |
| Fe | 0.011 | 0.001 | 0.732 | 7.719 | 0.000** |
| PTH | 0.000 | 0.000 | 0.062 | 2.081 | 0.038** |
| Ferritin | 0.000 | 0.000 | 0.122 | 3.613 | 0.000** |
| TIBC | -0.002 | 0.002 | -0.22 | -4..217 | 0.000** |
| A.S.T. [GOT] | -0.002 | 0.001 | -0.125 | -2.501 | 0.013** |
| Age | -0.004 | 0.001 | -0.158 | -5.282 | 0.000** |
| Year of treatment | -0.011 | 0.003 | -0.092 | -3.345 | 0.001** |
| W.B.C. | -0.023 | 0.006 | -0.115 | -3.798 | 0.000** |
| Tranferrin saturation | -0.027 | 0.003 | -0.847 | -8.134 | 0.000** |
| Hct | -0.048 | 0.017 | -0.529 | -2.869 | 0.004** |

***p<0.05 , **p<0.01**

**APPENDIX 3**

|  | Pearson product-movement correlation | Multiple regression | Decision Tree C5.0 | Logistic Regression | SVM Model  1 | SVM Model 2 | SVM Model  3 | SVM Model  4 | SVM Model 5 | percent |
| --- | --- | --- | --- | --- | --- | --- | --- | --- | --- | --- |
| Training sample group | | | | | | | | | | |
| Average Accuracy | --- | --- | 91.88% | 74.29% | 99.31% | 98.78% | 95.7% | 98.95% | 92.21% | --- |
| Standard Deviation | --- | --- | 2.16% | 0.66% | 0.24% | 0.45% | 0.68% | 0.39% | 0.8% | --- |
| Test sample group | | | | | | | | | | |
| Average Accuracy | --- | --- | 66.46% | 65.73% | 66.86% | 66.35% | 64.5% | 66.89% | 65.97% | --- |
| Standard Deviation | --- | --- | 4.21% | 1.59% | 2.44% | 1.59% | 2.26% | 2.2% | 1.9% | --- |
| Independent variables (40) | | | | | | | | | | |
| Age | ● | ● | ● | ● | ● | ● | ● | ● | ● | 100% |
| Type of fistula | ● | ● | ● | ● | ● | ● | ● | ● |  | 88.9% |
| Year of treatment | ● | ● | ● | ● |  | ● | ● |  | ● | 77.8% |
| Hemoglobin level (Hbc) | ● | ● | ● | ● | ● |  |  | ● | ● | 77.8% |
| Creatinine | ● | ● | ● | ● |  | ● | ● | ● |  | 77.8% |
| Calcium | ● | ● | ● | ● | ● |  | ● |  | ● | 77.8% |
| Hematocrit (Hct) | ● | ● | ● | ● | ● |  |  | ● |  | 66.7% |
| Total iron binding capacity (TIBC) | ● | ● | ● | ● |  |  |  | ● | ● | 66.7% |
| Post-dialysis weight | ● | ● | ● |  | ● | ● |  | ● |  | 66.7% |
| Gender | ● |  | ● |  | ● | ● |  | ● |  | 55.6% |
| Cholesterol level | ● |  | ● |  | ● | ● |  | ● |  | 55.6% |
| Mean corpuscular volume (MCV) | ● |  | ● | ● |  | ● | ● |  |  | 55.6% |
| Platelet count | ● |  | ● | ● | ● |  |  | ● |  | 55.6% |
| K | ● | ● | ● | ● |  |  |  |  | ● | 55.6% |
| White blood cell (WBC) count |  |  | ● | ● | ● | ● | ● |  |  | 55.6% |
| Aspartate aminotransferase(AST) | ● |  | ● | ● | ● |  |  |  |  | 44.4% |
| Triglyceride | ● |  | ● |  | ● | ● |  |  |  | 44.4% |
| Ferritin | ● |  | ● |  | ● | ● |  |  |  | 44.4% |
| Parathyroid hormone level (PTH) | ● |  | ● |  | ● | ● |  |  |  | 44.4% |
| Primary disease type |  |  |  | ● | ● |  | ● |  |  | 33.4% |
| Alanine aminotransferase (ALT) | ● |  | ● |  | ● |  |  |  |  | 33.3% |
| Glucose | ● |  | ● |  |  | ● |  |  |  | 33.3% |
| Transferrin saturation | ● |  | ● |  | ● |  |  |  |  | 33.3% |
| pre-dialysis body weight | ● |  | ● |  |  |  |  | ● |  | 33.3% |
| Post-dialysis blood urea nitrogen (BUN) level | ● |  |  | ● | ● |  |  |  |  | 33.3% |
| P | ● |  | ● |  |  |  |  | ● |  | 33.3% |
| Alkaline-P | ● |  |  |  |  |  |  | ● |  | 22.2% |
| R.B.C. |  |  | ● |  | ● |  |  |  |  | 22.2% |
| Weight loss from dialysis |  |  | ● |  |  | ● |  |  |  | 22.2% |
| Prior- dialysis  blood urea nitrogen (BUN) level | ● | ● |  |  |  |  |  |  |  | 22.2% |
| Uric acid | ● |  |  |  |  |  |  | ● |  | 22.2% |
| Normalized protein catabolic rate (NPCR) | ● |  | ● |  |  |  |  |  |  | 22.2% |
| Dehydration from ultrafiltration | ● |  |  |  |  |  |  | ● |  | 22.2% |
| Fe |  |  |  |  | ● |  |  |  |  | 11.1% |
| Urea reduction rate (URR) |  |  | ● |  |  |  |  |  |  | 11.1% |
| Kt/V(Gotch) | ● |  |  |  |  |  |  |  |  | 11.1% |
| TAC urea | ● |  |  |  |  |  |  |  |  | 11.1% |

**APPENDIX 4**

|  | Regularization parameters | | | | | | | | | |
| --- | --- | --- | --- | --- | --- | --- | --- | --- | --- | --- |
|  | 1 | | 10 | | 50 | | 100 | | 200 | |
| RBF  Gamma | Training | Test | Training | Test | Training | Test | Training | Test | Training | Test |
| 1 | 92.67% | 67.25% | 98.71% | 70.38% | 99.13% | 69.94% | 99.16% | 68.08% | 99.13% | 69.94% |
| 10 | 99.19% | 62.76% | 99.19% | 62.13% | 99.19% | 62.13% | 99.19% | 62.13% | 99.19% | 62.13% |
| 50 | 99.19% | 52.45% | 99.19% | 52.43% | 99.19% | 54% | 99.19% | 52.43% | 99.19% | 52.43% |
| 100 | 99.19% | 51.09% | 99.19% | 51.1% | 99.19% | 51.43% | 99.19% | 51.09% | 99.19% | 51.09% |
| 200 | 99.19% | 51.05% | 99.19% | 51.05% | 99.19% | 51.05% | 99.19% | 51.05% | 99.19% | 50.8% |

**APPENDIX 5**

| Actual  Prediction | Malnutrition | No Malnutrition |
| --- | --- | --- |
| Malnutrition | TP | FP |
| No Malnutrition | FN | TN |

**APPENDIX 6**

| Indicators | Equation |
| --- | --- |
| Accuracy | $\frac{TP+TN}{TP+FP+FN+TN}$ |
| Sensitivity | $\frac{TP}{TP+FN}$ |
| Specificity | $\frac{TN}{FP+TN}$ |
